# The Value of Prolactin, a Panel of Cytokines, and the Soluble Human Epidermal Growth Factor Receptor 2 in the Prediction of Rapid Progression and Shorter Survival during Palliative Chemotherapy of Colorectal Cancer Patients

**DOI:** 10.3390/biomedicines11072014

**Published:** 2023-07-18

**Authors:** Sylwia Cisoń-Jurek, Paulina Czajka-Francuz, Tomasz Francuz, Aleksander J. Owczarek, Bożena Szymczak, Jerzy Wojnar, Jerzy Chudek

**Affiliations:** 1 Department of Internal Medicine and Oncological Chemotherapy, Faculty of Medical Sciences in Katowice, Medical University of Silesia, Str. Reymonta 8, 40-027 Katowice, Poland; paulinaczajka@op.pl (P.C.-F.); bszymczak@sum.edu.pl (B.S.); jwojnar@sum.edu.pl (J.W.); 2 Department of Biochemistry, Faculty of Medical Sciences in Katowice, Medical University of Silesia, Str. Medyków 18, 40-752 Katowice, Poland; tfrancuz@mp.pl; 3 Health Promotion and Obesity Management Unit, Department of Pathophysiology, Faculty of Medical Sciences in Katowice, Silesian Medical University, Str. Medyków 18, 40-752 Katowice, Poland; aowczarek@sum.edu.pl

**Keywords:** colorectal cancer, cytokine, prolactin, palliative chemotherapy, chemokine, growth factors, predictive value, survival

## Abstract

The prediction of colorectal cancer (CRC) response to palliative chemotherapy (CTH) is still difficult. Patients at a higher risk of progression may benefit from more aggressive treatment. This study assessed the predictive value of prolactin (PRL) and a panel of cytokines, chemokines, and growth factors for the risk of rapid progression in CRC patients starting palliative CTH. This study included 51 CRC patients initiating palliative CTH with up to 5-year follow-up, divided into rapid and non-rapid progressors. Serum samples were collected before CTH for assessment of a large panel of cytokines, chemokines, growth factors, and PRL via a multiplex method. Rapid progressors (N = 19) were characterized by increased baseline values of IL-8 and IP10 but decreased PRL levels. In addition, PRL below 18.2 ng/mL was a strong predictor of weight loss during CTH. Grade 3 (HR = 2.97; 95%CI: 1.48–5.98) and PRL level (HR = 0.96; 95%CI: 0.91–1.01) were independent risk factors of progression. We showed that CRC rapid progressors are characterized by decreased baseline PRL levels. In addition, increased baseline levels of IP-10, sHER-2, IL-6, and IL-8 may be associated with longer survival; however, larger studies are needed to confirm their predictive role in CRC patients.

## 1. Introduction

A large body of evidence supports the involvement of cytokines, chemokines, and growth factors in all stages of carcinogenesis in colorectal cancer (CRC), the second most frequent contributor to cancer mortality [1]. Approximately 25% of patients with CRC have metastases at diagnosis, most commonly in the liver [2]. Late diagnosis, a high recurrence rate in locoregionally advanced disease despite adjuvant therapy, and aging of the population are followed by increasing CRC-related mortality worldwide.

New therapies allow for the personalization of treatment and improvement of outcomes with less toxicity in CRC patients. Subjects stratified as having a higher risk of rapid progression could be offered different therapies or more frequent follow-ups. Therefore, it would be of utmost importance to identify patients who are at higher risk of progression, before the therapy initiation, and offer a more aggressive approach.

However, there are currently limited tools to assess patients’ prognosis available to clinicians. Evidence of mutual connections between inflammation and carcinogenesis makes inflammatory cytokines, chemokines, and growth factors potentially attractive biomarkers as these compounds were found to modulate the immune response within the tumor microenvironment (TME).

The TME can suppress tumor growth via proinflammatory action or contribute to tumor progression via immunosuppression, angiogenesis, or facilitation of immune escape. Thus, defining a serum biomarker profile before the CRC systemic therapy may identify patients at risk of early recurrence or rapid progression, with this approach contributing to reductions in mortality [3].

The balance between pro- and anti-inflammatory various cytokines, chemokines, cytokine receptor expression as well as cancer and TME cells can be considered predictors of cancer response [4]. It was shown that the assessment of circulating interleukins may have prognostic significance in stage IV CRC patients [5]. On the other hand, only a few data regarding the predictive role of chemokines, growth factors, and hormones including prolactin for the CRC progression in stage IV patients have been published. Therefore, we extended additional data for these patients.

This study aimed to assess the predictive value of prolactin and a panel of cytokines, chemokines, and growth factors for the risk of rapid progression and survival in palliative chemotherapy of CRC patients.

## 2. Materials and Patients

### 2.1. Patients

This study involved 51 patients with advanced CRC (histologically confirmed adenocarcinomas), starting palliative CTH between May 2014 and October 2016 in a single oncology clinic. This prospective study design was accepted and approved by the Bioethics Committee (Approval no: KNW/0022/KB1/155/14). This study was conducted following the Declaration of Helsinki, and written informed consent was obtained from each subject. Inclusion criteria included pathologically confirmed colorectal adenocarcinoma, initially advanced or locally/locoregionally advanced after progression to metastatic disease, and qualified for palliative CTH. Among exclusion criteria were age below 18 years; planned anti-EGFR therapy; a known genetic predisposition for CRC, i.e., familial adenomatous polyposis (FAP); Peutz-Jeghers syndrome (PJS); juvenile polyposis syndrome or Lynch syndrome, a hereditary non-polyposis colorectal cancer (HNPCC); as well as acute or chronic infections; poor performance status (ECOG 3); history of other cancer; pregnancy; and breastfeeding. Clinical staging included an initial medical history, physical examination, colonoscopy with biopsy and pathological results, and imaging studies. All the patients completed at least one CTH cycle and were included in the analysis. Imaging before initiation of CTH included computed tomography (CT) or ultrasound examination of the abdomen, and chest radiographs or CT were applied depending on laboratory tests (CEA and Ca19.9) and at the physician’s discretion.

The first peripheral blood samples in fasting patients were obtained from subjects before the initiation of CTH.

Patients’ survival status was determined based on hospital and outpatient clinic records or phone calls if the patient discontinued clinical follow-up.

### 2.2. Assays

Blood samples were centrifuged at 300× *g* for 20 min, and then serum samples were stored in a liquid nitrogen vapor until assessment. Determinations of selected markers were performed in previously collected serum samples.

The profile of 35 cytokines, chemokines, and growth factors with a potential prognostic value in CRC patients was identified by the authors based on the literature analysis.

Serum levels of IL-1 beta, IL-1ra, IL-2, IL-4, IL-5, IL-6, IL-7, IL-8, IL-9, IL-10, IL-12(p70), IL-13, IL-17, FGF basic, G-CSF, GM-CSF, IFN-gamma, IP-10, MCP-1, MIP-1a, MIP-1b, TNF-alpha, VEGF, sEGFR, FGF-basic, sHER2/neu, HGF, sIL-6Ra, PDGF-AB/BB, PECAM-1, Prolactin, SCF, sTIE-2, sVEGFR-1, and sVEGFR-2 were measured via Multiplex technique Bio-Plex ProTM, in accordance with the manufacturer’s instruction, using kits from Bio-Rad. The bead fluorescence readings were taken using the Bio-Plex 200 System with the high PMT (High RP1) setting and analyzed with Bio-Plex Manager version 6.1.0.727 (Bio-Rad Laboratories, Hercules, CA, USA).

### 2.3. Data Analysis

The patients were categorized into 2 subgroups: rapid progressors and non-rapid progressors. Rapid progression was defined as progression according to RECIST criteria or a patient’s death due to disease progression not documented by visualization within 3 months of CTH. The clinical response for CTH in non-progressors was evaluated following clinical standards after 3 and 6 months and classified as partial or complete response or stabilization of the disease according to RECIST criteria. Characteristics of the study group was presented in Table 1.

### 2.4. Statistical Analysis

The STATISTICA 13.1 (TIBCO Software Inc., Palo Alto, CA, USA) and STATA 13.1 (StataCorp, Lakeway Drive, Texas, USA) served for data analysis. A *p*-value < 0.05 was set as the level of statistical significance with two sided-tests. No data imputation methods were used. Data with a normal distribution are presented as mean ± standard deviation and with a non-normal distribution or heavily skewed as median (lower quartile–upper quartile). Data on the nominal and interval scales are presented as counts and percentages. The Shapiro-Wilk test and the quantile-quantile (Q-Q) plot verified data distribution. The χ^2^ or the Fisher test for variables on the nominal and ordinal scale and the Student’s *t*-test for independent variables for data on the interval scale were used for data comparison. In the case of skewed data, a logarithmic normalization was done. The Cox proportional hazard analysis was used to assess risk factors for neutropenia. The results were presented as the hazard ratio (HR) with a corresponding 95% confidence interval (CI) and the value of statistical significance level. In each case, the fulfilment of the proportional hazard assumption was assessed based on the Schoenfeld residuals. The risk factors relevant to the univariable analysis were included in the multivariable analysis. Survival analysis was based on Kaplan-Meier curves, and their comparisons were made with the log-rank test.

## 3. Results

Characteristics of the whole study group and baseline values of selected routine assessed variables, as well as rapid and non-rapid progression subgroups, are presented in Table 1 and Table 2. The frequency of grade 1 and 2 adenocarcinomas was significantly higher in non-rapid progressors in comparison to rapid progressors. There were no differences in colon cancer location, surgery type, CTH regimens, and other parameters between mentioned subgroups.

Subsequently, we analyzed differences in PFS and OS between rapid and non-rapid progressors subgroups. The median PFS in the study group was 9.2 (3.8–19.7) months, and, of those, 37.3% developed rapid progression with a median PFS of 3.2 (1.6–4.7) months, while 62.7% were non-rapid progressors with a median of 17.0 (9.4–25.3) months. The median OS in the study group was 14.3 (7.3–28.6) months. Of those, 37.3% developed rapid progression with a median survival of 5.5 (3.2–11.7) months, while 62.7% were non-rapid progressors with a median OS of 24.3 (13.9–36.8) months. As a consequence of the stratification, PFS and OS were significantly longer in the non-rapid progressors subgroup (*p* < 0.001).

Next, we conducted a univariable analysis of baseline levels of cytokines, chemokines, and growth factors to find out their correlation with rapid progression. There were significantly increased baseline values of IL-8 and IP10, but decreased levels of PRL were found in the rapid progressors subgroup (Table 3). Other assessed cytokines, chemokines, and growth factors were not significantly different.

The mean body mass change during the 6-month follow-up was 1.9% (range: −14.4–21.0%), and a loss of weight was observed in 42.8% of patients. Of note, there was a significant correlation between log10(PRL) and the relative percentage change of body mass during the 6-month follow-up (r = 0.33; *p* < 0.05). Namely, the lower the baseline PRL level, the higher the weight loss. Based on the ROC curve, the cut-off level of PRL for weight loss was 18.2 ng/mL (AUC = 0.701; 95% CI: 0.534–0.868).

**Figure 1:**
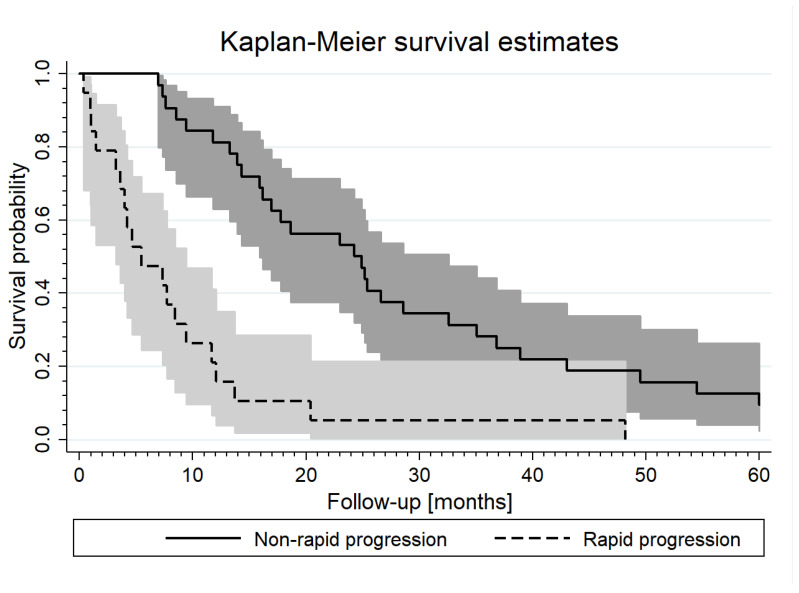
Kaplan–Maier curves estimate for rapid and non-rapid progressors (*p*_log-rank_ < 0.001).

Similarly, we also did a univariable analysis to assess if any of the baseline clinical parameters were associated with a higher risk of progression and death. The most important factor of progression was grade 3, following the location of cancer in the rectum and older age. In addition, rapid progression was the most important factor of death, following grade 3 and the location of cancer in the rectum and abdominoperineal resection. Moreover, the higher level of CEA, the higher risk of death (a tendency to statistical significance) (Table 4). Furthermore, it was shown that IL-6, IL-8, IP-10, sHER2, and PRL levels were significantly associated with the increased risk of both progression and death in the whole study group. In the multivariable survival analysis, the risk factor of progression and death were PRL level (HR = 0.96; 95% CI: 0.91–1.01; *p* = 0.09 and HR = 0.97; 95% CI: 0.92–1.02; *p* = 0.28, respectively) and grade 3 (HR = 2.97; 95% CI: 1.48–5.98; *p* < 0.01 and HR = 2.58; 95% CI: 1.25–5.33; *p* < 0.05, respectively). Moreover, rapid progression increased almost 4-fold the risk of death (HR = 3.60; 95% CI: 1.87–6.94; *p* < 0.001). Figure 1 shows the Kaplan–Maier curve estimates for rapid and non-rapid progressors groups.

## 4. Discussion

Our results indicate that low baseline prolactin and high IL-6, IL-8, IP-10, and sHER2 levels were associated with rapid progression and death in CRC patients receiving palliative CTH.

### 4.1. Predictive Value of Prolactin

The predictive role of prolactin in CRC has not been fully examined yet. In our study, we showed that increased pre-treatment PRL levels could be associated with longer OS in CRC patients receiving palliative CTH. Lower PRL baseline values were associated with rapid progression and an increased risk of death in CRC patients.

These results are contrary to previous studies, which found higher preoperative PRL levels in patients who developed progressive disease than in those who achieved response to treatment during 3 years of follow up [6,7,8]. In addition, several papers reported PRL levels correlated with the CRC stage [9,10]. However, previous studies included patients with different CRC stages, whereas our study included patients treated with palliative intent.

Of note, we showed that decreased PRL levels could be associated with the loss of weight as suggested in other clinical [11] and animal studies [12]. Indeed, loss of weight was observed in 42.8% of patients. Moreover, a significant correlation between log10(PRL) and the percentage change in body mass during the 6-month follow-up was found. This finding could at least partially explain lower levels of PRL in the rapid progressor group. This result suggests also that PRL could predict cancer-associated weight loss that frequently is associated with progressive disease.

It is worth mentioning that, via multiplex analysis, it is not possible to differentiate between full-length PRL (23 kDa) with demonstrated pro-tumor, proangiogenic activity, and 16 kDa isoform prolactin [13], which was proposed as an anti-tumor marker [14].

### 4.2. Predictive Value of IP-10 (IP-10, CXCL10)

The current study showed that baseline values of IP-10 showed a positive trend toward the rapid progression in CRC patients during CTH. Moreover, baseline IP-10 levels were significantly associated with the increased risk of progression and death in the whole study group.

Previously published studies indicated that IP-10 may play a dual role in cancer development. It can enhance anti-tumor immune response via supporting cytotoxic T-cell function and inhibiting angiogenesis, which can decrease tumor growth [15,16]. However, IP-10 can also act the opposite way, when autocrine secretion and CXCL10/CXCR3 signaling by cancer cells support tumor cell proliferation, angiogenesis, and metastasis [17], inhibiting cancer cells’ apoptosis and immune response. Circulating CXCL10 can stimulate cancer cell recruitment to bone and the formation of osteolytic bone metastasis [18]. It has been demonstrated that co-expression of IP-10 and its receptor, CXCR3, was associated with increased metastasis and with early progression as well as poor overall survival [19]. IP-10 modulates the function of cytotoxic T cells [20] and act as a chemotactic agent for NK and T cells, dendritic cells, macrophages, and B cells.

Other clinical studies also assessed the predictive value of IP-10. The authors confirmed the association of high serum IP-10 level and the risk of the development of metastasis but not with the recurrence rate in CRC patients during a 4-year follow-up. Moreover, the level of CXCL10 was significantly associated with mortality in these patients [21]. Our data further support the clinical significance of high IP-10 levels, showing the positive trend of IP-10 levels with the risk of rapid progression and death in CRC patients receiving palliative CTH.

### 4.3. Predictive Value of sHER2

While overexpression of human epidermal growth factor receptor 2 (HER2) has been documented in approximately 7% of cases of metastatic CRC [22] and is considered a negative predictor in metastatic CRC [23], the predictive significance of soluble HER2 (sHER2) has not been determined yet. Soluble HER2 is the extracellular domain of HER2 that can be cleaved by matrix metalloproteinases and released into circulation. An additional source of sHER could be alternate mRNA splicing [24]. While a high baseline sHER2 level can be considered a prognostic biomarker in HER2-positive breast cancer, the prognostic value of sHER in CRC is still not clear.

No clinical studies assessing predictive significance have been performed so far in CRC. Our study was the first to show that the sHER2 level was significantly associated with the increased risk of progression and death in CRC patients receiving palliative CTH.

### 4.4. Predictive Value of IL-8 and IL-6

Although the role of IL-8 in CRC tumor biology is well documented, the clinical significance of IL-8 in CRC prognosis has not been fully established yet. Other authors suggested that increased IL-8 levels may be a predictive factor of the progression and associated with worse survival in CRC [25].

IL-8 is one of the major pro-tumor factors in CRC. It promotes angiogenesis, proliferation, invasion, migration, and survival of CRC cells, engaged in metastasis formation and the epithelial–mesenchymal transition [26,27,28].

A recent meta-analysis of 12 clinical studies demonstrated that high serum IL-8 levels correlated with shorter OS and PFS in advanced CRC patients [29]. Moreover, it was confirmed that elevated level of IL-8 before treatment was correlated with progressive disease [30,31]. Our study supports that increased baseline values of IL-8 are associated with rapid progression and death by demonstrating a positive trend.

The predictive significance of IL-6 was confirmed in previous studies and a meta-analysis of 17 clinical studies by Xu et al. [32]. In line with previous findings, we showed that CRC patients before the initiation of palliative CTH with increased baseline IL-6 levels had a greater risk of progression and death.

IL-6 is one of the main pro-tumor cytokines, showing mainly immunosuppressive properties [33,34,35]. IL-6 is considered a growth factor for colon cancer cells; inhibition of IL-6 signaling slowed down the tumor cell’s growth [34]. It was demonstrated that IL-6 contributed to the acceleration of tumor progression and increased the migration of CRC cells [36].

The current study also confirmed the importance of clinical parameters for the risk of CRC progression including grade 3 adenocarcinoma diagnosis, the location of cancer in the rectum, and more advanced age. These parameters were also found in previous clinical studies in metastatic CRC patients [37].

Our study has several limitations. First, the study group was relatively small, which can be associated with a lack of significance for several analyzed factors. Moreover, HER2 status of the cancer tissue was not routinely assessed which may interfere with circulating sHER.

## 5. Conclusions

The identification of new prognostic factors could help in the identification of CRC patients with a high risk of rapid progression that may benefit from more aggressive anticancer systemic therapy. We showed that the rapid progressors are characterized by decreased baseline prolactin levels. In addition, increased baseline levels of IP-10, sHER-2, IL-6, and IL-8 may be associated with a longer survival time; however, larger studies are needed to confirm their predictive role in CRC patients receiving palliative chemotherapy.

## Figures and Tables

**Table 1:** Characteristics of patients starting palliative CTH with stratification to rapid and non-rapid progression subgroups at the end of the third month of treatment; *p*-value between progressors and non-progressors group.

	All	Non-Rapid Progression	Rapid Progression	*p*
N (%)	51 (100%)	32 (62.7)	19 (37.3)	
Female, N (%)	28 (54.9%)	20 (62.5%)	8 (42.1)	0.16
Age [years]	66 ± 9	64 ± 7	68 ± 10	0.11
BMI [kg/m^2^]	26.1 ± 4.5	26.4 ± 4.6	25.8 ± 4.5	0.66
WHO, N (%)				
0	37 (72.6)	25 (78.1)	12 (63.2)	0.51
1	12 (23.5)	6 (18.8)	6 (31.6)	
2	2 (3.9)	1 (3.1)	1 (5.2)	
Cancer location, N (%)				0.28
Right colon	13 (25.5)	7 (21.9)	6 (31.6)	
Left colon	12 (23.5)	9 (28.1)	3 (15.8)	
Sigmoid	13 (25.5)	10 (31.2)	3 (15.8)	
Rectum	13 (25.5)	6 (18.8)	7 (36.8)	
Clinical stage, N (%)				
II/III	16 (31.40	10 (31.2)	6 (31.6)	0.98
IV	35 (68.6)	22 (68.8)	13 (68.4)	
Grade, N (%)				
1 + 2	39 (76.5)	29 (90.6)	10 (52.6)	<0.01
3	12 (23.5)	3 (9.4)	9 (47.4)	
RAS mutation, N (%)	18 (75.0)	14 (73.7)	4 (80.0)	1.00
Surgery type, N (%)				
Hemicolectomy	17 (33.3)	9 (28.1)	8 (42.1)	0.06
Segmental resection	14 (27.4)	13 (40.6)	1 (5.3)	
Lower anterior resection	12 (23.5)	6 (18.8)	6 (31.6)	
Colostomy	8 (15.7)	4 (12.5)	4 (21.0)	
Radiation therapy, N (%)	12 (23.53)	7 (21.9)	5 (26.3)	0.72
Chemotherapy, N (%)				
Initially palliative	23 (45.1)	15 (46.9)	8 (42.1)	0.74
Palliative after radical therapy	28 (54.9)	17 (53.1)	11 (57.9)	
5-FU monotherapy	11 (21.6)	5 (15.6)	6 (31.6)	0.55
FOLFIRI	31 (60.8)	21 (65.6)	10 (52.6)	
FOLFOX-4	7 (13.7)	5 (15.6)	2 (10.5)	
FOLFOX4+bevacizumab	2 (3.9)	1 (3.1)	1 (5.3)	

Mean ± standard deviation or number (percentage).

**Table 2:** Baseline values of selected routine-assessed variables in rapid and non-rapid progression subgroups; *p*-value between progressors and non-progressors group.

	All Patients [N = 51]	Non-Rapid Progressors [N = 32]	Rapid Progressors [N = 19]	*p*
Hemoglobin [g/dL]	12.2 ± 1.6	12.4 ± 1.6	11.8 ± 1.6	0.26
Red blood cells [10^6^/μL]	4.34 ± 0.45	4.38 ± 0.49	4.28 ± 0.38	0.46
White blood cells [10^3^/μL]	7.7 ± 3.2	7.8 ± 3.2	7.6 ± 3.2	0.86
Platelets [10^3^/μL]	291 ± 133	300 ± 125	276 ± 147	0.53
CEA [ng/mL]	22.8 (4.8–100.8)	29.5 (4.8–92.1)	20.4 (5.3–335.5)	0.99
Ca19-9 [U/mL]	15.2 (7.2–92.2)	31.9 (7.2–122.0)	11.1 (5.8–40.3)	0.26
AST [IU/L]	23 (16–39)	21 (14–34)	24 (17–44)	0.16
ALT [IU/L]	21 (16–37)	20 (15–38)	21 (16–27)	0.84

Mean ± standard deviation or median (lower quartile–upper quartile).

**Table 3:** Differences in baseline concentrations of assessed cytokines, chemokines, and growth factors in rapid and non-rapid progressors subgroups; *p*-value between progressors and non-progressors group.

Parameter	Non-Rapid Progressors [N = 32]	Rapid Progressors [N = 19]	*p*
IL-8 [pg/mL]	19.8 (15.6–29.3)	21.7 (16.6–44.4)	0.08
IP-10 [pg/dL]	6.6 (4.4–81.3)	7.4 (6.2–103.4)	0.08
PRL [ng/mL]	19.5 (13.6–50.7)	17.9 (10.0–29.4)	<0.05

Median (lower quartile–upper quartile).

**Table 4:** Univariable analysis of baseline clinical parameters for the risk of progression and death in the whole study group. The significant values are bold.

	Progression	
	HR	±95% CI
Male vs. Female	1.04	0.59–1.85
Age [per decade]	**1.49 ^$^**	1.00–2.22
BMI [per kg/m^2^]	0.99	0.93–1.05
WHO 1/2 vs. 0	1.54	0.82–2.89
Cancer location		
Right colon	1.65	0.72–3.80
Left colon	0.86	0.37–2.00
Sigmoid	Ref.	
Rectum	2.88 *	1.23–6.76
Clinical Stage VI vs. II/III	1.14	0.61–2.12
Grade 3 vs. 1/2	**3.28 ^#^**	1.64–6.59
RAS mutation	1.22	0.45–3.33
Surgery type		
Hemicolectomy	Ref.	
Segmental resection	0.64	0.30–1.37
Lower anterior resection	1.60	0.75–3.42
Abdominoperineal resection	1.62	0.68–3.86
Radiation therapy	1.15	0.58–2.26
Chemotherapy		
Initially palliative	Ref.	
Palliative after radical therapy	0.84	0.47–1.51
Rapid progression	–	–
Initial values of:		
CEA [per ng/dL]	1.02	0.98–1.07
Ca19-9 [per U/dL]	1.03	0.83–1.28
Baseline values		
IL-6 [per pg/mL]	1.27 *	1.03–1.56
IL-8 [per 10 pg/mL]	1.10 **	1.03–1.17
IP-10 [per pg/dL]	1.08 *	1.01–1.16
sHER2 [per ng/mL]	1.05 *	1.01–1.09
PRL [per 10 ng/mL]	**0.95 ^$^**	0.90–1.00

Ref.—reference group, HR—hazard ratio, CI—confidence interval; ^$^ *p* < 0.1, * *p* < 0.05, ** *p* < 0.01, ^#^ *p* < 0.001.

## Data Availability

The data presented in this study are available on request from the corresponding author. The data are not publicly available due to subject privacy restrictions.
